# Selenium Nanoparticles as Potential Antioxidants to Improve Semen Quality in Boars

**DOI:** 10.3390/ani13152460

**Published:** 2023-07-30

**Authors:** Pavel Horky, Lenka Urbankova, Iqra Bano, Tomas Kopec, Pavel Nevrkla, Magdalena Pribilova, Daria Baholet, Pompido Chilala, Petr Slama, Sylvie Skalickova

**Affiliations:** 1 Department of Animal Nutrition and Forage Production, Faculty of AgriSciences, Mendel University in Brno, Zemedelska 1, 613 00 Brno, Czech Republic; urbankoval@centrum.cz (L.U.); xpribilo@mendelu.cz (M.P.); daria.baholet@mendelu.cz (D.B.);; 2 Department of Physiology and Biochemistry, Faculty of Bio-Sciences, Shaheed Benazir Bhutto University of Veterinary and Animal Sciences, Sakrand, Nawabshah 67210, Pakistan; iqrashafi05@yahoo.com; 3 Department of Animal Breeding, Faculty of AgriSciences, Mendel University in Brno, Zemedelska 1, 613 00 Brno, Czech Republic; tomas.kopec@mendelu.cz (T.K.); pavel.nevrkla.uchhz@mendelu.cz (P.N.); 4 Laboratory of Animal Immunology and Biotechnology, Department of Animal Morphology, Physiology and Genetics, Mendel University in Brno, 61300 Brno, Czech Republic; petr.slama@mendelu.cz

**Keywords:** nanoparticles, selenium, semen, antioxidants, boars

## Abstract

**Simple Summary:**

Duroc boars were supplemented by sodium selenite or selenium nanoparticles at the dose of 0.3 mg Se/kg/day for 126 days. Sampling was carried out at days 0, 42, 84 and 126. Sperm qualitative parameters and antioxidant activity were compared with those of the control group (without selenium supplementation). The findings indicate that SeNPs slightly improved GPx activity, and both SeNPs and sodium selenite influenced the antioxidant capacity of semen. This research also showed that both treatments did not affect semen qualitative parameters based on comparisons with the control group.

**Abstract:**

Selenium is an essential compound which can influence the fertility of boars by a greater margin. In past decades, research was mainly focused on a bioavailability of various selenium forms and the effect on semen quality. Recently, nanotechnology has expanded the possibilities of selenium supplementation research. Twenty-one Duroc boars (three groups with seven boars each) were included in this experiment with the first group being a control group with no selenium supplementation, and the second group being supplemented with 0.3 mg Se/kg of selenium in inorganic form of Na_2_SeO_3_. The third group was supplemented with selenium nanoparticles (100 nm) at the same dose as that of the second group. The experiment lasted for 126 days (three spermatogenesis cycles of boars) and the antioxidant parameters of boar semen were analysed at 42, 84 and 126 days, respectively. The antioxidant parameters (DPPH, FRAP, DMPD, GSH, GSSG) were not influenced by both Se_2_NO_3_ and selenium nanoparticle supplementation during this experiment. At the end of the monitored period, significantly higher (*p* < 0.004) antioxidant readings were observed by using the ABTS method but not the DPPH, DMPD and FRAP methods on the supplemented groups compared to the control. Moreover, selenium-nanoparticle-supplemented groups showed elevated glutathione peroxidase activity in the seminal fluid (*p* < 0.008). However, the selenium nanoparticle supplementation has not shown an improving effect on sperm quality. This could be considered as a safe alternative to inorganic selenium as well as having a potential to enhance the antioxidant properties of the semen of boars.

## 1. Introduction

Intensive pig production has increased demand on husbandry and nutrition in piggeries around the world. In particular, the nutrition of pigs of all categories has been carefully studied for a long time. The breeding of boars requires a specific feeding strategy to maintain the quality and quantity of the semen [1]. Recently, it has been shown that an antioxidant-rich diet has beneficial effects on semen quality because it helps the body to cope with free radicals that arise during spermatogenesis [2]. Oxidative stress is linked to poor sperm function, morphology, and motility; it is also considered one of the causes of DNA damage [3,4]. The main antioxidant defence of spermatogenesis includes enzymes such as superoxide dismutase, glutathione peroxidase, glutathione-S-transferase. These antioxidants are involved in the rapid conversion of superoxide anions to hydrogen peroxide. Generated hydrogen peroxide is eliminated by catalase or glutathione peroxidase (GPx). In addition, non-enzymatic antioxidants play a pivotal role in antioxidant defence, and the most important of them are the components of antioxidant enzymes: zinc, selenium, vitamins C and E, glutathione, carotenoids, melatonin, and cytochrome C [2]. 

Selenium (Se) is the main component of selenoenzymes involved in sperm protection against oxidative stress. Besides its antioxidant roles, selenium has also been proven to be essential for spermatogenesis, biosynthesis of testosterone, sertoli cell function as well as morphological and functional parameters of sperms [5]. In addition, Se has been linked to the improvement of semen quality during cryopreservation [5]. Recently, the boar seminal plasma macro- and microelements were observed to be associated with boar sperm quality, and this provides new information on sperm quality after it has been stored in a liquid form. It is possible to estimate sperm quality after storage using fresh boar seminal plasma selenium concentrations due to high correlations between fresh seminal plasma and semen characteristics after storage [6]. 

Chemical forms of selenium and its bioavailability have been an interesting research question in past decades with the main motivation for these investigations being the narrow distinction between toxicity and therapeutic dose. It is now well established from a variety of studies that the organic form of Se has better bioavailability and is less toxic when compared to the inorganic form. However, with the development of nanotechnologies, new forms of nano-sized Se have been investigated. Extensive research in this area has shown that Se nanoparticles (SeNPs) are more bioavailable and have a wide therapeutic window [7]. In terms of antioxidant capacity, several studies have confirmed their higher efficiency to scavenge free radical species [8,9,10]. 

Selenium nanoparticles have been studied as a supplement in pig nutrition in terms of antioxidant properties and oxidative stress prevention which could influence the overall growth performance [8]. The suggested mechanism of action of SeNPs antioxidant activity is associated to activity enhancement of selenoenzymes such as the glutathione peroxidase family and thioredoxin reductase compared to inorganic and organic selenium forms [11]. However, the antioxidant effect of SeNPs relies on their method of synthesis or surface modification. It has been shown that the green synthetised SeNPs have generally higher antioxidant capacity compared to chemically synthesised SeNPs in vitro. Thus, the selection of a SeNP form depends on its purpose. For the enhancement of enzymatic antioxidant defence, it is crucial to synthetise SeNPs with gradual and effective release of selenium ions [12]. 

In this pilot experiment, the influence of SeNPs and inorganic selenium in the form of sodium selenite on stud boar semen and antioxidant parameters was investigated. The treatment period 126 days was chosen due to the boar spermatogenesis cycle, which takes 42 days. We hypothesised that changes could be manifested after three cycles.

## 2. Materials and Methods

### 2.1. Chemicals

2.2-diphenyl-1-picrylhydrazyl (DPPH), 2.2′-azino-bis (3-ethylbenzothiazoline-6-sulfonic acid (ABTS), N,N-dimethyl1,4-diaminobenzene (DMPD), reduced glutathione, oxidised glutathione, and other chemicals were purchased from Sigma-Aldrich unless otherwise stated. SeNPs (99.99% purity, size: <100 nm) were purchased from Nanografi (Ankara, Turkey). The reagents were freshly prepared for each day and used immediately. Stock standard solutions (1 mg/mL) were prepared with ACS water (Sigma-Aldrich, Prag, Czech Republic) and stored at −20 °C in the dark. Working standard solutions were prepared daily by diluting the stock solutions. The pH value was measured using WTW inoLab Level 3 with terminal Level 3 (WTW GmbH, Weilheim, Germany).

### 2.2. Animals 

The experiment was carried out in the sperm collection centre of boars in Velke Mezirici (Czech Republic). The experiment included 21 boars of the Duroc breed. The average age was 2 ± 0.3 years, and the average weight of the boars was 250 ± 20 kg. The experimental animals were individually housed (2.5 × 2.5 m) with the ad libitum access to water. All animals were fed with 3.3 kg of basic feed mixture (Table 1). The content of metabolised energy (ME_p_) was a 12.6 MJ/kg diet. The boars were divided into three groups (n = 7). These boars were reared according to the standards for the Duroc breed [13]. The control group had no increased levels of selenium in their diet. The second group was supplemented with sodium selenite at the dose of 0.3 mg of Se/kg feed. The third experimental group was supplemented by SeNPs at the dose of 0.3 mg Se/kg feed. The characterisation of the used selenium nanoparticles can be found in our previously published study [14]. 

The experimental observations lasted for 126 days (three spermatogenesis cycles of boars) and boar semen was collected once a week. For biochemical analysis, the semen was taken at the beginning of the experiment (Day 0), and in the 42, 84 and 126 days of this experiment. The semen was collected by a gloved hand method using the breeding phantom. The aliquot of the samples for antioxidant activity assays were frozen and stored at −20 °C prior to analysis. 

### 2.3. Determination of Volume, Sperm Concentration and Motility and Percentage of Abnormal Sperm in Semen

The values of semen volume, motility, sperm concentration and percentage of abnormal sperm were analysed. The sampling of semen was carried out at Day 0 (before treatment), Day 42, Day 84 and Day 126 of the experiment. 

Semen quality determination was performed according to the methodology of Lovercamp et al. [15]. The concentrations of semen were evaluated using a self-calibrating photometer (SpermaCueTM, Minitube of America, Verona, WI, USA). The motility analyses were performed using the Sperm VisionTM software (Minitube of America, Verona, WI, USA) on images obtained by a digital camera attached to a phase contrast microscope (Olympus microscope IX 71 S8F-3; Tokyo, Japan). Prior to analysis, 500 µL from each fresh sample was diluted with 500 µL of the Androhep extender and incubated at 37 °C for 30 min. The evaluation of sperm morphology and cellular particles was performed using a phase contrast microscope (Zeiss; West Germany). The subjective analyses were always carried out by the same qualified person. 

### 2.4. Sample Preparation 

Firstly, a 0.5 mL volume of thawed semen was pipetted followed by the addition of 2 mL of liquid nitrogen and 0.5 mL of a 100 mM phosphate buffer (pH 6.5). Subsequently, the samples were homogenised in a homogeniser, ULTRA-TURRAX T8 (IKA, Konigswinter, Germany), at 3000 rpm for 2 min. After the homogenisation, 1 mL of a phosphate buffer was added to each sample. The samples were centrifuged in a Universal 32 R centrifuge (Hettich-Zentrifugen GmbH, Tuttlingen, Germany) at 16,000 rpm at 4 °C for 20 min. Finally, the supernatant was removed and used for the analyses (1.5 mL).

### 2.5. Determination of the Antioxidant Properties of Semen

Assays were carried out on an automated biochemical analyser BS200 (Mindray, Shenzhen, China) according to [16]. The absorbance difference from 2 min and 10 min were used for calculating the results. The results were calculated from the freshly prepared calibration curves (gallic acid) and evaluated by software (Mindray, China). Inter-assay-CVs of the methods were 6% and 8%, respectively (n = 20). The assay methods are listed below.

### 2.6. DPPH Test

In total, 150 µL of the reagent (0.095 mM 2,2-diphenyl-1-picrylhydrazyl—DPPH^•^ in HPLC grade 100% methanol) was incubated with 15 µL of the sample. Absorbance was measured at 505 nm for 10 min. 

### 2.7. Ferric Reducing Antioxidant Power (FRAP)

A 150 μL FRAP reaction mixture was injected into a plastic cuvette with a subsequent addition of a 3 μL sample. Absorbance was measured at 605 nm for 10 min. 

### 2.8. ABTS Test

A total in 150 µL of 7 mM 2,2′-azinobis-3-ethylbenzothiazoline-6-sulfonic acid (ABTS^•^) and 4.95 mM potassium peroxodisulphate were mixed with 3 µL of the sample. Absorbance was measured at 660 nm for 10 min. 

### 2.9. DMPD Assay

Reagent preparation was carried out in the following ways: Solution 1: 0.2 mM of acetate buffer (pH 5.2). Solution 2: 0.74 mM of ferric chloride. Solution 3: 36.7 mM of DMPD. Solutions 1, 2 and 3 were mixed in a 20:1:1 (*v*/*v*/*v*) ratio. Procedure: A total of 160 μL of reagent was injected into a plastic cuvette with the subsequent addition of a 4 μL sample. Absorbance was measured at 505 nm. 

### 2.10. GSH and GSSG Measurement

GSH and GSSG analyses were carried out on an HPLC-ED system (ESA, Inc., Chelmsford, MA, USA). A chromatographic column with reverse phase Zorbax eclipse AAA C18 (Agilent Technologies, Inc., Santa Clara, CA, USA; 150 × 4.6 mm; 3.5 μm particles) was used. A total of 20 μL of the sample was injected using an autosampler thermostated at 35 °C. Mobile phase A consisted of a 100% methanol and mobile phase B consisted of an aquaeolus 80 mM trifluoracetic acid. Oxidised and reduced glutathione were eluted by increasing the linear gradient as follows: 0–1 min (3% B), 1–2 min (10% B), 2–5 min (30% B) and 15–16 min (98% B). The flow rate was set at 1 mL/min. The electrochemical detector was set on a potential of 900 mV.

### 2.11. Determination of Glutathione Peroxidase

A glutathione peroxidase cellular activity assay kit (CGP1, Sigma Aldrich, St. Louis, MO, USA) was used for the GPx assessment, and determination was performed in native semen. For the determination of glutathione peroxidase activity, the BS 400 automated spectrophotometer (Mindray, China) was utilised. The experimental protocol was prepared according to the following parameters: reagent R1 (0.3 mM NADPH in GPx buffer) of 260 µL in volume was pipetted into a plastic cuvette with the subsequent addition of 10 µL of sample. After mixing, 30 µL of reagent R2 (3 mM tert-butyl hydroperoxide) was added to the cuvette, which started the reaction. The decrease in absorbance was measured at 340 nm using the kinetic program for 126 s.

### 2.12. Determination of Selenium Concentration 

Differential pulse voltammetry (797 VA Computrace, Metrohm, Herisau, Switzerland) was used for Se analysis. The three-electrode system consisted of components described further. Working: a hanging mercury drop electrode (drop area of 0.4 mm^2^); reference: an Ag/AgCl/3M KCl electrode; auxiliary: a glassy carbon electrode. The parameters of the measurement were as follows: deposition potential of −0.6 V, accumulation time of 200 s, pulse amplitude of 0.03 V, pulse time of 0.05 s, voltage step of 0.006 V, voltage step time of 0.1 s, sweep rate of 0.06 V/s, equilibration time of 30 s. The scan was in the range of potentials of −0.4 V to −0.9 V and the characteristic peak of selenium was recorded at a potential of −0.7 V. The total volume of the measuring vessel was 2 mL (1980 µL of electrolyte and 20 µL of sample). The electrolytes consisted of a 15 µM ammonium sulphate (pH 2.2) with the addition of copper sulphate (0.05 mM *v*/*v*). The pH was adjusted using sulfuric acid. 

### 2.13. Statistics

Statistical analysis was performed using statistical program R [17]. The evaluation of the influence of “Form” and “Sampling” on boar semen parameters (DPPH, GSH, GSSG, GPX, Se, and volume of semen concentration of sperms) was carried out using the analysis of variance and the post hoc analysis Scheffé’s test. The distribution of residual errors was approximately normal. Non-parametric analysis of variance (the Kruskal–Wallis test) was used for the properties of FRAP, ABTS, DMPD, motility and abnormal sperms. The null hypothesis was rejected in all cases at the significance level α = 0.05.

## 3. Results

**Figure 1:**
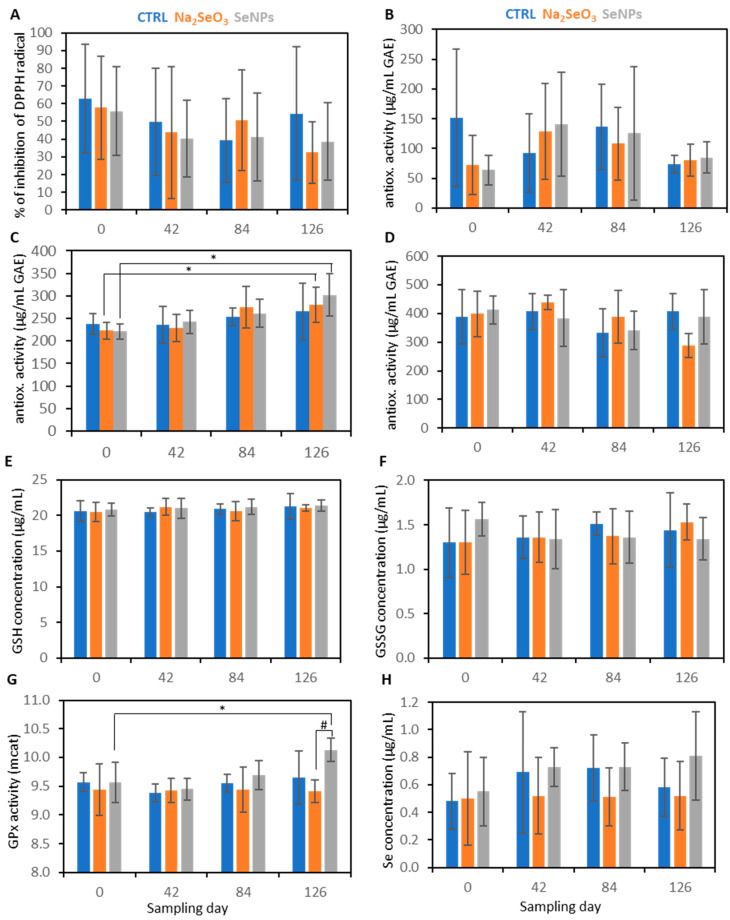
Biochemical parameters of semen. (**A**) DPPH, (**B**) FRAP, (**C**) ABTS, (**D**) DMPD, (**E**) GSH, (**F**) GSSG, (**G**) GPx and (**H**) selenium. Semen was analysed at Day 0 (without any treatment), and Days 42, 84 and 126. Boars were supplemented with sodium selenite or SeNPs at the dose of 0.3 Se mg/kg/day. Values are expressed as a mean ± SD (n = 7). Asterisks show significance (*p* < 0.05) of the results compared to the control group (Day 0). Hashtag shows significance (*p* < 0.05) of the results between sampling days.

The total antioxidant activity of the semen was analysed using four methods. As shown in Figure 1A,B,D, no significant differences between the control group and supplemented groups using sodium selenite and SeNPs were observed by the DPPH, DMPD and FRAP methods. Compared to the sampling Day 0, a significantly increased (*p* < 0.004) total antioxidant capacity analysed by using the ABTS method was observed in sodium selenite and SeNP supplemented groups at Days 84 and 126 of sampling (Figure 1C). No statistically significant differences between the control and supplemented groups were observed for GSH and GSSG. SeNP supplementation significantly (*p* < 0.008) increased GPx activity at the end of the experiment compared to those of Day 0 and sodium selenite supplementation (Figure 1G). No statistical significance was observed in selenium concentrations in the semen of boars. 

There was no evidence that both sodium selenite and SeNPs supplementation improved semen quality parameters (Table 2).

## 4. Discussion

A strong relationship between selenium supplementation and semen quality among animal species has been reported in the literature [18]. Selenium nanoparticles are a promising chemical form of selenium which shows better bioavailability and decreased toxicity compared to inorganic or organic selenium at the same dose [19]. In this experiment, the influence of SeNPs on antioxidant capacity and semen quality parameters were evaluated. The results were compared with those of the control group without any selenium supplementation and the group of boars supplemented with inorganic selenium (Na_2_SeO_3_). Antioxidant parameters tested by DPPH, DMPD, and FRAP methods have not been improved by SeNP supplementation compared to inorganic selenium and control groups. Only the ABTS method has shown significant improvement in the antioxidant capacity of SeNP- and inorganic-selenium-treated groups at Day 126 of sampling. In addition, the activity of GPx was increased at the same time, but only in the SeNP group. Other parameters such as GSH, GSSG and selenium concentration in semen did not show significant improvement. The semen quality parameters were slightly improved in the SeNP-treated group; however, the differences were not significant [20].

The unanticipated result was where the ABTS was the only procedure confirming a significant improvement in antioxidant activity with inorganic Se and SeNP supplementation. These results could be the outcome of the different principles and reaction environments of the involved methods. All of them use synthetically formed radicals which are scavenged by antioxidants, and the antioxidant activity is expressed and quantified by colour change [21]. Compared to the DPPH method, the ABTS reaction is in an aqueous environment with physiological pH which is the most natural for semen. DPPH uses organic solvents such as methanol or ethanol. The DMPD assay requires an acidic environment, and the results are not reproductive for hydrophobic antioxidants. Common antioxidant capacity assays are the FRAP method which is based on Fe^3+^ reduction in a low-pH environment. However, the main disadvantage of this method is different reaction time for antioxidant species [22]. For this reason, it is suggested that these methods cannot be compared, and moreover, the antioxidant activity should not be accepted based on a single antioxidant testing procedure [23]. However, monitoring the antioxidant capacity of semen seems to be an important parameter because some findings support the idea that the antioxidant capacity of seminal plasma correlates with the abnormal sperm occurrence and the sperm quality of boars coupled with functionality [24,25].

In this study, the effect of SeNP supplementation is not intended directly as an antioxidant but is a co-factor of preventive antioxidant enzyme GPx which reduces H_2_O_2_ to H_2_O in the mammalian cells. Simultaneously, the cycle of oxidation of and reduction in glutathione takes place. Our results found that an increase in GPx activity occurred only after 126 days of supplementation and only in the SeNP-supplemented group. It seems possible that these results are explained by a later onset of the selenium effect at the administered dose. The findings of the current study are not fully supported by the results of the previous research of Petrujkić et al. who found an increased GPx activity in boar seminal plasma after 90 days of supplementation at the dose of 0.3 mg/kg of sodium selenite [25]. In contrast, Pavaneli et al. did not observe the effects of dietary Se treatments at the same doses on GPx activity in seminal plasma [26]. 

Previous studies have explored the relationships between selenium supplementation and semen quality. However, the reported research findings are inconsistent. This study confirms that selenium in both forms (inorganic and nano) did not significantly improve semen qualitative parameters. Similar results have been obtained by Lovercamp et al. who supplemented a basal diet with 0.3 ppm of selenium in inorganic and organic forms [15]. In contrast, earlier findings showed that organic selenium at 0.3 ppm improved sperm motility and antioxidant parameters [25]. It can therefore be concluded that the effect of selenium depends on its form and the duration of action. 

In many studies, a debate has taken place regarding inorganic and organic selenium concerning their bioavailability and toxicity [27,28,29]. Thus far, no relevant research has been conducted on the effect of SeNP supplementation on semen qualities of boars, and for this reason, the results are compared with those of other species of livestock. In a previous study dating back to 2016, the influence of vitamin E and SeNPs on the antioxidant status and semen quality of roosters was investigated. The higher sperm motility (increased by 10.4% to 25.2%), the antioxidant parameters (increased by 105%), and the GPx activity (increased by 14.6%) were observed in the groups supplemented with vitamin E and SeNPs. Moreover, a significantly decreased concentration of malondialdehyde was observed [30]. Interesting results were obtained in the experiment on older roosters, and it is well known that aging worsens semen quality. SeNPs (10–40 nm) at the dose of 0.3 mg Se/kg diet maintained sperm motility after 5 weeks of supplementation compared to the control group supplemented with inorganic forms of Se at the same dose [31]. The effect of SeNPs on the semen quality of buck goats was studied with a diet containing a dose of 0.3 mg Se/kg, which had no effect on semen volume, but abnormal sperm count was higher. These results are not in agreement with the results of the present study, where the count of abnormal sperms did not show a significant increase compared to the control group. On the contrary, the GPx activity was significantly higher in the SeNP-supplemented group compared to the control group, which supports our results [32]. Wistar albino rats supplemented with SeNPs (40–60 nm) at a dose of 0.5 mg Se/kg failed to significantly improve in sperm motility and abnormal sperm occurrence, but GPx activity and total antioxidant activities were significantly increased compared to those of the control group [20]. These results are consistent with the data obtained in our findings from the boars which were under study. Higher activity of GPx in blood was also observed in sows [33], piglets [34] and fattening pigs [8] after SeNP enrichment of their diet.

These results draw our attention to the importance of considering selenium supplementation for boars. In particular, farmers should evaluate the benefits of modest increases in glutathione peroxidase and antioxidant activity at the cost of selenium or selenium nanoparticles. The results from other researchers, when they only examined the benefits of selenium in inorganic and organic form, are also contradictory. However, the combination of selenium and other antioxidants such as vitamin E appears to be promising, as we published earlier [35]. In addition, these findings are also supported by results of other studies [36,37]. Selenium or SeNPs, as such, are suitable to be added before cryopreservation, because they have demonstrably improved effects on the quality of semen after thawing [36,38]. 

## 5. Conclusions

This study set out to assess the effects of inorganic selenium and SeNP supplementation at a dose of 0.3 mg/kg/diet on a boar semen quality and its antioxidant parameters. The findings indicated that SeNPs slightly improved GPx activity, and both SeNPs and sodium selenite influenced the antioxidant capacity of semen, which was confirmed by the ABTS method. On the other hand, changes in the antioxidant activity, in comparison to the control group, were not proven by DPPH, FRAP, and DMPD methods. In addition, the GSH:GSSG ratio did not show significant changes in the treated groups. The research also showed that sodium selenite and SeNP supplementation did not affect the qualitative parameters of semen compared to the control group. The evidence from this study suggests that the addition of SeNPs to the diet of breeding boars does not significantly improve the quality of the ejaculate. Therefore, supplementation should be carefully considered in terms of feed costs. This paper contributes to recent discussions concerning the effectiveness of SeNPs as a novel form of selenium supplementation. Further research should focus on determining synergies between SeNPs and other antioxidants which seem to be promising tools for enhancing the quality of boar semen.

## Figures and Tables

**Table 1:** The composition of the feed mixture for boars.

Component	% in Feed Mixture
Barley grain	36
Wheat grain	20.36
Oat grain	20
SBM (soybean meal)	14.5
EKPO T	3
BergaFat	2.1
Calcium carbonate	1.5
Monodicalciumphosphate	1.2
Mineral vitamin premix for boars	0.5
Sodium chloride	0.4
Magnesium oxide	0.15
L-Lysine HCl	0.14
L-Threonine	0.09
Methionine DL	0.06

Bergafat (Berg + Schmidt, Germany)—palm oil; EKPO T (Delika—Pet, Czech Republic)—biscuit meal.

**Table 2:** Qualitative and quantitative traits of semen at Day 0 (without any treatment) and Days 42, 84, 126. Boars were supplemented by sodium selenite or SeNPs at the dose of 0.3 Se mg/kg/day. Data are presented as means and standard deviations of analysis (n = 7).

	Control				Na_2_SeO_3_				SeNPs				
	Day of Sampling				Day of Sampling				Day of Sampling				*p*-Value
Parameter	0	42	84	126	0	42	84	126	0	42	84	126	
Volume (mL)	120 ± 14	159 ± 60	126 ± 26	144 ± 51	145 ± 30	126 ± 27	167 ± 58	155 ± 16	161 ± 59	108 ± 23	135 ± 37	93 ± 17	0.662
Motility (%)	75 ± 5	74 ± 5	69 ± 2	74 ± 5	72 ± 5	72 ± 5	69 ± 2	66 ± 7	71 ± 4	73 ± 5	72 ± 5	75 ± 5	0.106
Count (bill.)	670 ± 200	694 ± 212	607 ± 109	752 ± 146	647 ± 148	672 ± 135	740 ± 153	681 ± 144	675 ± 141	750 ± 178	851 ± 113	810 ± 163	0.332
Abnormal sperms (%)	13 ± 2.1	9.9 ± 3.3	12 ± 4.5	9.4 ± 2.7	12 ± 4.9	16 ± 11.9	14 ± 4.8	17.0 ± 11.2	12 ± 2.3	16 ± 9.8	10.0 ± 2.4	8.6 ± 4.6	0.255

## Data Availability

The datasets used and/or analysed during the current study are available from the corresponding author on reasonable request.

## References

[1] Rodriguez A.L., Van Soom A., Arsenakis I., Maes D. (2017). Boar management and semen handling factors affect the quality of boar extended semen. Porc. Health Manag..

[2] Kowalczyk A. (2022). The Role of the Natural Antioxidant Mechanism in Sperm Cells. Reprod. Sci..

[3] Agarwal A., Virk G., Ong C., du Plessis S.S. (2014). Effect of Oxidative Stress on Male Reproduction. World J. Mens Health.

[4] Wagner H., Cheng J.W., Ko E.Y. (2018). Role of reactive oxygen species in male infertility: An updated review of literature. Arab J. Urol..

[5] Qazi I.H., Angel C., Yang H.X., Zoidis E., Pan B., Wu Z.Z., Ming Z., Zeng C.J., Meng Q.Y., Han H.B. (2019). Role of Selenium and Selenoproteins in Male Reproductive Function: A Review of Past and Present Evidences. Antioxidants.

[6] Pipan M.Z., Mrkun J., Strajn B.J., Vrtac K.P., Kos J., Pislar A., Zrimsek P. (2017). The influence of macro- and microelements in seminal plasma on diluted boar sperm quality. Acta Vet. Scand..

[7] Zambonino M.C., Quizhpe E.M., Mouheb L., Rahman A., Agathos S.N., Dahoumane S.A. (2023). Biogenic Selenium Nanoparticles in Biomedical Sciences: Properties, Current Trends, Novel Opportunities and Emerging Challenges in Theranostic Nanomedicine. Nanomaterials.

[8] Zheng Y.L., Dai W.Z., Hu X.L., Hong Z.P. (2020). Effects of dietary glycine selenium nanoparticles on loin quality, tissue selenium retention, and serum antioxidation in finishing pigs. Anim. Feed Sci. Technol..

[9] Horky P., Ruttkay-Nedecky B., Nejdl L., Richtera L., Cernei N., Pohanka M., Kopel P., Skladanka J., Hloucalova P., Slama P. (2016). Electrochemical Methods for Study of Influence of Selenium Nanoparticles on Antioxidant Status of Rats. Int. J. Electrochem. Sci..

[10] Lee M.T., Lin W.C., Yu B., Lee T.T. (2017). Antioxidant capacity of phytochemicals and their potential effects on oxidative status in animals—A review. Asian-Australas. J. Anim. Sci..

[11] Bisht N., Phalswal P., Khanna P.K. (2022). Selenium nanoparticles: A review on synthesis and biomedical applications. Mater. Adv..

[12] Sentkowska A., Pyrzynska K. (2022). The Influence of Synthesis Conditions on the Antioxidant Activity of Selenium Nanoparticles. Molecules.

[13] Council N.R. (2012). Nutrient Requirements of Swine: Eleventh Revised Edition.

[14] Urbankova L., Skalickova S., Pribilova M., Ridoskova A., Pelcova P., Skladanka J., Horky P. (2021). Effects of Sub-Lethal Doses of Selenium Nanoparticles on the Health Status of Rats. Toxics.

[15] Lovercamp K.W., Stewart K.R., Lin X., Flowers W.L. (2013). Effect of dietary selenium on boar sperm quality. Anim. Reprod. Sci..

[16] Sochor J., Pohanka M., Ruttkay-Nedecky B., Zitka O., Hynek D., Mares P., Zeman L., Adam V., Kizek R. (2012). Effect of selenium in organic and inorganic form on liver, kidney, brain and muscle of Wistar rats. Cent. Eur. J. Chem..

[17] R Development Core Team (2022). R: A Language and Environment for Statistical Computing.

[18] Surai P.F., Fisinin V.I. (2015). Selenium in Pig Nutrition and Reproduction: Boars and Semen Quality—A Review. Asian-Australas. J. Anim. Sci..

[19] Skalickova S., Milosavljevic V., Cihalova K., Horky P., Richtera L., Adam V. (2017). Selenium nanoparticles as a nutritional supplement. Nutrition.

[20] Hozyen H.F., Khalil H.M.A., Ghandour R.A., Al-Mokaddem A.K., Amer M.S., Azouz R.A. (2020). Nano selenium protects against deltamethrin-induced reproductive toxicity in male rats. Toxicol. Appl. Pharmacol..

[21] Karadag A., Ozcelik B., Saner S. (2009). Review of Methods to Determine Antioxidant Capacities. Food Anal. Methods.

[22] Amorati R., Valgimigli L. (2015). Advantages and limitations of common testing methods for antioxidants. Free Radic. Res..

[23] Gulcin I. (2020). Antioxidants and antioxidant methods: An updated overview. Arch. Toxicol..

[24] Barranco I., Tvarijonaviciute A., Perez-Patino C., Parrilla I., Ceron J.J., Martinez E.A., Rodriguez-Martinez H., Roca J. (2015). High total antioxidant capacity of the porcine seminal plasma (SP-TAC) relates to sperm survival and fertility. Sci. Rep..

[25] Petrujkic B.T., Sefer D.S., Jovanovic I.B., Jovicin M., Jankovic S., Jakovljevic G., Beier R.C., Anderson R.C. (2014). Effects of commercial selenium products on glutathione peroxidase activity and semen quality in stud boars. Anim. Feed Sci. Technol..

[26] Pavaneli A.P.P., Martinez C.H.G., Nakasone D.H., Pedrosa A.C., Mendonca M.V., Martins S., Kawai G.K.V., Nagai K.K., Nichi M., Fontinhas-Netto G.V. (2021). Hydroxy-selenomethionine as an organic source of selenium in the diet improves boar reproductive performance in artificial insemination programs. J. Anim. Sci..

[27] Au A., Mojadadi A., Shao J.Y., Ahmad G., Witting P.K. (2023). Physiological Benefits of Novel Selenium Delivery via Nanoparticles. Int. J. Mol. Sci..

[28] Bhattacharjee A., Basu A., Bhattacharya S. (2019). Selenium nanoparticles are less toxic than inorganic and organic selenium to mice in vivo. Nucleus.

[29] Constantinescu-Aruxandei D., Frincu R.M., Capra L., Oancea F. (2018). Selenium Analysis and Speciation in Dietary Supplements Based on Next-Generation Selenium Ingredients. Nutrients.

[30] Safa S., Moghaddam G., Jozani R.J., Kia H.D., Janmohammadi H. (2016). Effect of vitamin E and selenium nanoparticles on post-thaw variables and oxidative status of rooster semen. Anim. Reprod. Sci..

[31] Alavi M.H., Allymehr M., Talebi A., Najafi G. (2020). Comparative effects of nano-selenium and sodium selenite supplementations on fertility in aged broiler breeder males. Vet. Res. Forum.

[32] Shi L.G., Yang R.J., Yue W.B., Xun W.J., Zhang C.X., Ren Y.S., Shi L., Lei F.L. (2010). Effect of elemental nano-selenium on semen quality, glutathione peroxidase activity, and testis ultrastructure in male Boer goats. Anim. Reprod. Sci..

[33] Horky P., Skalickova S., Urbankova L., Baholet D., Kociova S., Bytesnikova Z., Kabourkova E., Lackova Z., Cernei N., Gagic M. (2019). Zincphosphate-based nanoparticles as a novel antibacterial agent: In vivo study on rats after dietary exposure. J. Anim. Sci. Biotechnol..

[34] Liu C.L., Li Y.F., Li H.Y., Wang Y.C., Zhao K. (2022). Nano-Selenium and Macleaya cordata Extracts Improved Immune Functions of Intrauterine Growth Retardation Piglets under Maternal Oxidation Stress. Biol. Trace Elem. Res..

[35] Horky P., Skladanka J., Nevrkla P., Slama P. (2016). Effect of Diet Supplemented with Antioxidants (Selenium, Copper, Vitamins E And C) on Antioxidant Status and Ejaculate Quality of Breeding Boars. Ann. Anim. Sci..

[36] Gloria A., Contri A., Grotta L., Carluccio A., Robbe D., Ianni A., Vignola G., Martino G. (2019). Effect of dietary grape marc on fresh and refrigerated boar semen. Anim. Reprod. Sci..

[37] Pena S.T., Gummow B., Parker A.J., Paris D. (2019). Antioxidant supplementation mitigates DNA damage in boar (*Sus scrofa domesticus*) spermatozoa induced by tropical summer. PLoS ONE.

[38] Dorostkar K., Alavi-Shoushtari S.M., Mokarizadeh A. (2012). Effects of in vitro selenium addition to the semen extender on the spermatozoa characteristics before and after freezing in water buffaloes (*Bubalus bubalis*). Vet. Res. Forum.

